# HTLV-1 burden dependent on hijacking monocytes chemotaxis

**DOI:** 10.1186/1742-4690-12-S1-P7

**Published:** 2015-08-28

**Authors:** Maria F Castro-Amarante, Cynthia A Pise-Masison, Katherine McKinnon, Robyn W Parks, Veronica Galli, Raya Massoud, Giovanna Brunetto, Breanna Caruso, David Venzon, Steven Jacobson, Genoveffa Franchini

**Affiliations:** 1 Animal Models and Retroviral Vaccines Section, National Cancer Institute, Bethesda, MD, USA; 2 Viral Immunology Section, Neuroimmunology Branch, National Institute of Neurological Disorders and Stroke, Bethesda, MD, USA; 3 Biostatistics and Data Management Section, National Cancer Institute, Bethesda, MD, USA

## 

The HTLV-DNA burden in PBMCs is a risk factor for HAM/TSP and ATL development. We investigated the contribution of monocyte subsets (classical, intermediate and non-classical) to the total viral burden in 23 HTLV-1 infected individuals by assessing their frequency, their chemotactic and phagocytic functions, as well as their infectivity status. Classical monocytes differed between infected and uninfected individuals, their frequency was lower and their expression level of the chemokine receptors CCR5, CXCR3 and CX3CR1 was higher. While the percentage and surface chemokine receptor expression did not differ between HTLV-1 infected and uninfected individuals, intermediate monocytes from HTLV-1 infected individuals had increased migratory capacity to CCL5, the ligand for CCR5. Non-classical monocytes from HTLV-1 infected individuals increased in frequency and expressed high levels of CCR1, CXCR3 and CX3CR1. All three purified monocyte subsets were infected by HTLV-1. The level of viral DNA in monocyte subsets correlated directly with their migration capacity to CCL2, CCL5 and CX3CL1 for the classical subset, with lesser phagocytosis for the intermediate monocytes, and with the level of viral DNA in CD8+ and CD4+T-cells for the non-classical subset. These data suggest a model whereby HTLV-1 infection augments classical monocytes migration to tissues resulting in their infection, and non-classical monocytes frequency, resulting in increased transmission of virus to CD4+ and CD8+T-cells. Our data in humans together with prior animal experiments supports the notion that infection of monocytes in vitro is crucial for viral infectivity and persistence in humans, rendering infected monocytes desirable therapeutic targets.

